# Effect of synbiotic supplementation on obesity and gut microbiota in obese adults: a double-blind randomized controlled trial

**DOI:** 10.3389/fnut.2024.1510318

**Published:** 2024-11-27

**Authors:** Xiaokang Niu, Qi Zhang, Julong Liu, Yuyang Zhao, Nan Shang, Shusen Li, Yinghua Liu, Wei Xiong, Erna Sun, Yong Zhang, Hongfeng Zhao, Yixuan Li, Pengjie Wang, Bing Fang, Liang Zhao, Juan Chen, Fuqing Wang, Guofang Pang, Chenyuan Wang, Jingjing He, Ran Wang

**Affiliations:** ^1^Key Laboratory of Functional Dairy, Co-Constructed by Ministry of Education and Beijing Government, Department of Nutrition and Health, China Agricultural University, Beijing, China; ^2^Research Center for Probiotics, China Agricultural University, Beijing, China; ^3^Mengniu Hi-Tech Dairy Product Beijing Co., Ltd., Beijing, China; ^4^Department of Nutrition, The First Medical Center of Chinese PLA General Hospital, Beijing, China; ^5^Food Laboratory of Zhongyuan, Luohe, China; ^6^Tibet Tianhong Science and Technology Co., Ltd., Lhasa, China; ^7^Chinese Academy of Inspection and Quarantine, Beijing, China

**Keywords:** *Bifidobacterium animalis* subsp. *lactis* MN-Gup, synbiotics, obesity, gut microbiota, randomized controlled trial

## Abstract

**Background:**

Synbiotics, combining specific probiotics and selected prebiotics, may benefit health issues like obesity, but evidence remains inconsistent.

**Objective:**

This study aimed to verify the effect of a pre-screened synbiotics combination [containing *Bifidobacterium animalis* subsp. *lactis* MN-Gup (MN-Gup), galacto-oligosaccharides (GOS) and xylo-oligosaccharides (XOS)] on obesity in the population.

**Methods:**

In a randomized, double-blind, placebo-controlled trial, 80 individuals with obesity consumed daily synbiotics (containing MN-Gup 1 × 10^11^ CFU/day, GOS 0.7 g/day, and XOS 0.7 g/day) or placebo for 12 weeks. Body composition, blood lipids, serum hormone, bile acids, and gut microbiota were measured pre-and post-intervention.

**Results:**

Synbiotics supplementation significantly decreased body fat percentage, waist, and serum low-density lipoprotein cholesterol (LDL-C), increased peptide YY, cholecystokinin, oxyntomodulin, GSH (glutathione peroxidase) in individuals with obesity. Additionally, synbiotic supplementation led to an enrichment of beneficial bacteria and bile acids chenodeoxycholic acid (CDCA). *Bifidobacterium* and *Romboutsia* were significantly positively correlated with CDCA. A more favorable effect was observed in individuals with obesity and abnormal LDL-C compared to those without dyslipidemia.

**Conclusion:**

Twelve-week synbiotics intervention reduced body fat percentage, waist, and serum LDL-C, especially in individuals with obesity and abnormal LDL-C. The possible mechanisms may be related to changes in gut microbiota, bile acids and gut hormones.

**Clinical trial registration:**

Chictr.org.cn, identifier ChiCTR2200064156.

## 1 Introduction

Obesity represents a serious global public health challenge, with its prevalence escalating rapidly over the past five decades, affecting approximately 700 million adults worldwide (1). Due to excessive fat accumulation, obesity is strongly associated with dyslipidemia, hypertension and cardiovascular diseases (2). There are currently various ways to alleviate obesity, such as drug intervention, surgical intervention, etc., but these methods have their own limitations, such as high side effects and high risks. Therefore, safe and effective ways to alleviate obesity are of great significance and need to be found.

Probiotics have garnered significant attention in the application of relieving obesity due to their potential in regulating gut microbiota and its metabolites (3–7). Research has shown that certain beneficial bacteria can modify bile acid composition (8). Additionally, secondary bile acids have been shown to active Takeda G protein-coupled receptor 5 (TGR5), thereby promoting the proliferation of intestinal epithelial cells (9), which subsequently secrete hormones such as peptide YY (PYY), cholecystokinin (CCK), oxyntomodulin (OXM), and human angiopoietin-like protein 4 (ANGPTL4) (10–12), all of which have been supported to possess the potential of inhibiting fat synthesis (13–15).

Prebiotics are organic substances that are not digested and absorbed by the host, but can selectively promote the proliferation of some bacteria in human gut, thereby improving the health of the host (16, 17). Besides, they can also increase the survival rate of probiotics in the gastrointestinal tract (18). The synergistic combinations of probiotics and prebiotics, known as synbiotics, are hypothesized to exert a more potent impact compared to individual probiotics and prebiotics (19). Prebiotics such as inulin, fructo-oligosaccharides (FOS), galacto-oligosaccharides (GOS), xylo-oligosaccharides (XOS), and polydextrose are commonly combined with probiotics to form synbiotics, aiming to enhance their effects (20–23). However, the efficacy of different synbiotics in improving obesity varies. For instance, a combination of *Lactobacillus acidophilus*, *Bifidobacterium lactis*, *Bifidobacterium longum*, and *Bifidobacterium bifidum* and GOS was found to regulate gut microbiota and reduce weight in individuals with obesity (24). Synbiotics containing *Bifidobacterium lactis* UBBLa-70 and FOS not only improved body composition but also regulated glucose and lipid metabolism (25). Conversely, another study showed that the synbiotics comprising *Lactobacillus acidophilus*, *Bifidobacterium lactis*, *Bifidobacterium longum*, and *Bifidobacterium bifidum* and GOS did not exhibit statistically significant differences in body composition (24).

Given that the primary objective of incorporating prebiotics in synbiotics is to selectively enhance the proliferation of probiotics, the formulation combining probiotics and prebiotics holds significant importance. The variability observed in the effect of synbiotics on ameliorating obesity could potentially be attributed to whether a pre-screening process for the combination of probiotics and prebiotics was conducted. Our previous *in vitro* pre-screening experiments have demonstrated that GOS and XOS exhibit significant potential in promoting the proliferation of *Bifidobacterium animalis* subsp. *lactis* MN-Gup (MN-Gup, which is from BaMa longevity village, Guangxi, China, CGMCC No. 15578), rendering them suitable for synergistic combining to form synbiotics combination (26). Furthermore, our subsequent animal experiments have revealed that MN-Gup-based synbiotics possessed promising capabilities in significantly alleviating obesity in mice (26). This study aimed to investigate the effect of synbiotics (MN-Gup-GOS-XOS, containing MN-Gup, GOS and XOS) on improving obesity-related indicators and gut microbiota through a randomized controlled trial involving individuals with obesity.

## 2 Methods

### 2.1 Study design

A randomized, double-blind, placebo-controlled trial was designed to investigate the effects of synbiotics on alleviating obesity in individuals with obesity in Beijing, China, and was performed between November 2022 and February 2023. The study was approved by the Institutional Review Board of China Agricultural University Ethics Committee (CAUHR-20220903, registered on September 23, 2022). Written informed permission was acquired from each individual.

### 2.2 Inclusion and exclusion criteria

Inclusion criteria: (1) aged 18–45 years; and (2) body mass index (BMI) ≥28 kg/m^2^ or body fat percentage for males >25%, females >30%.

Exclusion criteria: (1) secondary obesity caused by diseases (e.g., hypothalamic obesity, pituitary obesity, thyroid obesity, adrenal obesity, or pancreatic obesity); or (2) in pregnancy or lactation; or (3) suffering from severe gastrointestinal diseases (i.e., ulcers, bleeding, obstruction, tumors, etc.); or (4) with a history of other serious disorders (i.e., diseases of heart, liver, kidney, brain, hematopoietic system, mental, tumors, etc.); or (5) taking weight-loss pills, antibiotics, proton pump inhibitors, H_2_ receptor antagonists, laxatives, antidiarrheal drugs, antibacterial agents, and hormones within 4 weeks; or (6) had taken probiotics within 1 month before the study; (7) participated in other clinical trials within 3 months.

### 2.3 Randomization and blinding

The randomization process was conducted by a research assistant who was not involved in the study. Randomization was performed using the Pocock and Simon minimization dynamic randomization method, incorporating baseline data (age, gender, BMI, and body fat percentage) to ensure balanced allocation across intervention groups at baseline participants were randomly assigned (1:1) to the synbiotic group and placebo group. Researchers and participants were blinded to intervention assignments until the study was completed.

### 2.4 Intervention and procedures

Prior to the study, participants were recruited via the internet and were subjected to age, gender, body composition, disease status, and drug use for screening. This study included a 2-week run-in period and a 12-week intervention period (Supplementary Figure S1). Throughout the run-in period, the participants were strictly prohibited from consuming foods containing probiotics (such as probiotic powder, probiotic yogurt, etc.). During the intervention period, everyone was instructed to consume a sachet of synbiotics (containing MN-Gup 1 × 10^11^ CFU/day, GOS 0.7 g/day, and XOS 0.7 g/day) or a sachet of placebo (maltodextrin powder without probiotics and prebiotic) once daily after either lunch or dinner for a duration of 12 weeks. The synbiotics and placebo utilized in this study were provided by Mengniu Hi-Tech Dairy Product Beijing Co., Ltd. Participants were requested to maintain their regular diet and exercise routine and were instructed to refrain from taking any other probiotics or probiotic-containing dietary supplements. Any adverse events or discomfort experienced by the participants were to be noted down in their diaries. Blood and fecal samples were collected and body composition were detected before and after intervention, respectively.

### 2.5 Primary outcomes

Primary outcomes are BMI and body fat percentage (body fat mass/weight), which were measured using the Inbody 770 body composition tester (Biospace Shanghai Co., Ltd., China).

### 2.6 Exploratory outcomes

#### 2.6.1 Body composition measurement

Soft lean mass of subjects was measured using the Inbody 770 body composition tester (Biospace Shanghai Co., Ltd., China). Waist circumference and hip circumference (cm) were measured using a flexible tape.

#### 2.6.2 Blood sample collection and blood index measurement

Blood samples were collected via venipuncture following an overnight fasting period both before and after the intervention. Subsequently, serum was collected by centrifugation to quantify blood lipids, serum hormones, bile acids, as well as markers of liver and kidney function.

Total cholesterol (TC), triglycerides (TG), low-density lipoprotein cholesterol (LDL-C), and high-density lipoprotein cholesterol (HDL-C) were measured using Elabscience ELISA kits (Elabscience Biotechnology Co., Ltd., China). Hormone indicators including peptide YY (PYY), cholecystokinin (CCK), oxyntomodulin (OXM), angiopoietin like protein 4 (ANGPTL4), GSH (glutathione peroxidase), T-AOC (total antioxidant capacity) of serum were measured using Meimian ELISA kits (Jiangsu Meimian industrial Co., Ltd., China), according to the manufacturer’s instructions. Serum uric acid (UA), creatinine (CR), urea (UR), aspartate aminotransferase (AST), and alanine aminotransferase (ALT), as the safety indicators, were measured using COIBO ELISA kits (Shanghai COIBO Biotechnology Co., Ltd., China).

### 2.7 Dietary information measurement

A dietary questionnaire was employed to assess the individuals’ dietary information for three consecutive days, both before and after the intervention. Subjects were given written and verbal instructions on how to maintain a complete food record with sufficient detail for analysis. If information was incomplete, the staff contacted the individual to confirm details of the foods consumed. Energy, carbohydrate, fat, and protein intake were computed using the food exchange method (27).

### 2.8 Serum bile acid measurement

Fasting plasma samples were obtained via venipuncture and serum was collected by centrifugation. Serum bile acids were measured refers to the previous research method using Agilent ultra-high-performance liquid chromatography-mass spectrometry (UHPLC-MS) [Agilent Technologies (China) Co., Ltd., China], and is modified (28). The specific steps are as follows: 50 μL serum and 200 μL internal standard solution [consisting of lithocholic acid (LCA)-D4, DCA-D4, and glycochenodeoxycholic acid (GCDCA)-D4 in methanol] were added to a 1.5 mL centrifuge tube. After vortex oscillation (Haimen Kylin-Bell Lab Instruments Co., Ltd., China) and centrifugation [Eppendorf (Shanghai) International Trade Co., Ltd., China] at 4°C for 30 s, 200 μL supernatant was transferred to a new 1.5 mL centrifuge tube. Then 200 μL ultrapure water was added for freeze drying using Sihuan freeze dryer (Beijing Sihuan Qihang Technology Co., Ltd., Beijing) and the residue is redissolved in 160 μL ultra-pure water. The dissolved sample was centrifuged at 12,000 r/min for 5 min, and the supernatant was transferred to a liquid injection bottle for HPLC analysis. The chromatographic column is the ZORBAX RRHD C18 column of the Agilent 1290LC system (50 mm × 2.1 mm, 1.8 μm) [Agilent Technologies (China) Co., Ltd., China]. The mobile phase elution procedure was as follows: 0–3 min: 35–50% methanol, 3–7.5 min: 50–75% methanol, 7.5–12 min: 75–100% methanol, 12–14 min: 100% methanol. The injection volume, flow rate, and the column temperature were 2 μL, 0.4 mL/min, and 30°C, respectively. Mass spectrum conditions: spray voltage: 4,500 V, nitrogen temperature: 350°C, flow rate: 11 L/min, capillary voltage: 4,000 V in positive ion mode and 3,500 V in negative ion mode.

### 2.9 Gut microbiota measurement

Fresh fecal samples were collected at baseline and after 12 weeks of intervention, and were collected and stored in sterile retention bottles. The samples were promptly placed on ice, transported to the laboratory within 1 h, and then frozen at −80°C for future use. Gut microbiota analysis of fresh feces was completed by Shanghai Majorbio Bio-pharm Technology Co., Ltd. (Shanghai, China). Fecal samples were taken out from-80°C for thawing until they restored to room temperature. Fecal DNA were extracted according to the requirements of the kit [E.Z.N.A.^®^ SoilDNA, Annoron (Beijing) Biotechnology Co., Ltd., China]. Nanodrop micro spectrophotometer [Thermo Fisher Scientific (China) Co., Ltd., China] was used for DNA quality inspection. The hypervariable region V3–V4 of the bacterial 16S rRNA gene was amplified with primer pairs 338F (5′-ACTCCTACGGGAGGCAGCAG-3′) and 806R (5′-GGACTACHVGGGTWTCTAAT-3′). Sequencing was performed using the Miseq PE 300/NovaSeq PE 250 platform (Illumina, United States). Pathway information was obtained according to the KEGG database while the abundance of each functional category was calculated according to OTU abundance (29).

### 2.10 Statistical analysis

The sample size was estimated according to Stenman et al. (23) with the results of the change of body fat percentage and 39 subjects per group were needed with an *α*-error of 0.05 and *β*-error of 0.20. Considering the dropout rate, 40 individuals were planned to be recruited into each group.

Intent to treatment set (ITT) was used for analysis in this study. In the ITT set, missing values were imputed based on the last observation carried forward method. Continuous variables with normal or approximate normal distribution are described by mean ± standard deviation, whereas continuous variables with skewed distribution are described by median and interquartile range. Paired sample *t*-test and paired sample rank sum test were used for intra-group comparisons. Independent *t*-test and Mann–Whitney test were used for inter-group comparisons, and chi square test for counting data.

In the analysis of gut microbiota, the UPARSE algorithm was utilized for performing operational taxonomic unit (OTU) clustering analysis, while the RDP classifier algorithm was applied for taxonomic analysis. A principal coordinates analysis (PCoA) was conducted using unweighted-UniFrac distances of the OTUs to analyze β diversity. Differential abundance analysis of taxa was executed using linear discriminant analysis effect size (LEfSe). Correlation analysis was conducted using Spearman correlation analysis.

Due to LDL-C being a risk factor for multiple cardiovascular diseases, we conducted subgroup analysis on individuals with baseline LDL-C abnormalities and those without dyslipidemia. (Subgroup1: obese individuals with abnormal LDL-C; subgroup2: obese individuals without dyslipidemia) as the exploratory analysis (30).

*p* < 0.05 indicates a statistically significant difference. SAS 9.4 was used for statistical analysis.

## 3 Results

### 3.1 Characteristics of participants

This randomized, double-blind, placebo-controlled study was performed between November 2022 and February 2023. A total of 113 subjects were recruited for screening, out of which 80 subjects were included based on the inclusion criteria (Supplementary Figure S2). The subjects were randomly divided into a synbiotic or placebo group, with 40 subjects in each group. Thirteen subjects discontinued the intervention, leaving 67 subjects finally finishing the trial. At baseline, there were no statistically significant differences (*p* > 0.05) in age, sex, BMI, body fat percentage, waist circumference, hip circumference, soft lean mass between the two groups (Table 1). The final ITT analysis incorporated all 80 individuals who were randomized.

**Table 1:** Subject characteristics at baseline.

	Placebo (*n* = 40)	Synbiotics (*n* = 40)	*p*
Age	34 (25, 38)	29 (22, 37)	0.21
Sex (male: female) (%)	27.50: 72.50	30.00: 70.00	1.00
BMI (kg/m^2^)	26.84 (24.85, 30.72)	28.35 (24.25, 31.50)	0.48
Body fat percentage (%)	36.40 ± 4.12	37.12 ± 6.89	0.70
Waist (cm)	88.00 (79.00, 96.00)	89.50 (75.00, 98.50)	0.73
Hip (cm)	102.33 ± 8.43	104.60 ± 8.72	0.32
Soft lean mass (%)	58.74 ± 3.57	59.25 ± 6.57	0.93

BMI, body mass index. *p* indicates the differences between groups.

### 3.2 Effect of synbiotics intervention on body composition and serum indicators of individuals with obesity

As demonstrated in Table 2, the synbiotic group exhibited a significant decrease in BMI (*p* = 0.01), body fat percentage (*p* = 0.001), waist (*p* = 0.0001), and serum LDL-C level (*p* = 0.005) after the intervention, while no significant changes were observed in the placebo group. Moreover, the decrease in body fat percentage (−0.36% vs. −1.26%) and waist (−0.40 cm vs. −2.28 cm) in the synbiotic group was significantly greater than that in the placebo group. Besides, there was a significant inter-group difference regarding the changes of LDL-C (−0.19 mmol/L vs. 0.00 mmol/L, *p* = 0.03). Both groups exhibited remarkable increase in serum PYY (*p* < 0.01) and OXM (*p* < 0.001) levels. However, the synbiotic group showed significantly higher levels of PYY (*p* = 0.006) and OXM (*p* = 0.0002) compared to the placebo group after intervention, with a particularly notable inter-group difference in the change of OXM (*p* = 0.02). Substantial increases in serum CCK (*p* = 0.0006) and ANGPTL4 (*p* = 0.04) was observed in the synbiotic group, but not the placebo group, with a significantly higher serum CCK compared to the placebo group following the synbiotic intervention (*p* = 0.03). As for antioxidant indexes, after intervention, the serum GSH levels of both groups significantly increased (*p* < 0.01), but the GSH levels in the synbiotics group were significantly higher than those in the placebo group after intervention (*p* = 0.0001), and the increase value was significantly greater than that in the placebo group (*p* = 0.005). There was no significant change in T-AOC between the two groups before and after intervention.

**Table 2:** Effect of synbiotics intervention on body composition of individuals with obesity.

	Placebo group				Synbiotic group					
	V0	V1	Change	*p*_0_	V0	V1	Change	*p*_0_	*p*_1_	*p*_2_
BMI (kg/m^2^)	26.84 (24.85, 30.72)	26.45 (24.79, 28.81)	−0.11 ± 0.65	0.43	28.35 (24.25, 31.50)	28.05 (23.70, 31.20)	−0.32 ± 0.75	0.01	0.51	0.17
Body fat percentage (%)	36.40 ± 4.12	36.04 ± 4.23	−0.36 ± 1.65	0.21	37.12 ± 6.89	35.86 ± 7.30	−1.26 ± 1.66	0.001	0.89	0.02
Waist (cm)	88.00 (79.00, 96.00)	86.00 (79.00, 93.00)	−0.40 ± 4.44	0.56	89.50 (75.00, 98.50)	87.00 (73.50, 99.00)	−2.28 ± 3.55	0.0001	0.78	0.02
Hip (cm)	102.33 ± 8.43	100.86 ± 9.01	−1.47 ± 3.88	0.03	104.60 ± 8.72	102.23 ± 8.32	−2.38 ± 2.83	0.0001	0.50	0.25
Soft lean mass (%)	58.74 ± 3.57	59.75 ± 4.07	0.89 ± 0.15	0.006	59.25 ± 6.57	60.49 ± 6.95	1.21 ± 1.56	0.0001	0.45	0.76
TG (mmol/L)	1.31 ± 1.04	1.31 ± 0.67	−0.01 ± 0.89	0.96	1.55 ± 1.05	1.59 ± 1.10	0.05 ± 0.79	0.33	0.19	0.75
TC (mmol/L)	4.63 ± 0.88	4.72 ± 1.04	0.05 ± 0.45	0.47	4.80 ± 0.86	4.76 ± 0.86	−0.04 ± 0.50	0.61	0.84	0.39
LDL-C (mmol/L)	3.01 ± 0.72	3.03 ± 0.84	0.00 ± 0.35	0.99	3.12 ± 0.59	2.94 ± 0.57	−0.19 ± 0.39	0.005	0.58	0.03
HDL-C (mmol/L)	1.23 ± 0.25	1.18 ± 0.24	−0.04 ± 0.15	0.09	1.17 ± 0.27	1.12 ± 0.25	−0.06 ± 0.16	0.11	0.32	0.85
PYY (pg/mL)	231.67 ± 23.58	246.65 ± 23.31	15.14 ± 29.59	0.005	239.67 ± 18.71	262.23 ± 21.72	22.57 ± 29.02	0.0001	0.006	0.31
CCK (ng/L)	333.40 ± 26.35	341.08 ± 28.57	8.64 ± 33.96	0.15	332.58 ± 25.49	356.36 ± 26.69	23.78 ± 35.12	0.0006	0.03	0.08
OXM (pg/mL)	2716.78 ± 251.29	2990.24 ± 296.53	288.18 ± 322.17	0.0001	2773.56 ± 209.43	3254.08 ± 249.45	480.53 ± 324.71	0.0001	0.0002	0.02
ANGPTL4 (ng/mL)	39.52 ± 3.38	41.09 ± 3.82	1.61 ± 5.09	0.07	39.88 ± 3.75	41.96 ± 3.69	2.08 ± 5.35	0.04	0.35	0.72
GSH (ng/mL)	61.45 ± 227.30	63.27 ± 5.76	2.06 ± 7.76	0.0002	61.32 ± 5.27	68.67 ± 4.89	7.35 ± 6.82	0.0001	0.0001	0.005
T-AOC (ng/mL)	4.05 ± 0.72	4.06 ± 0.95	−0.01 ± 1.06	0.95	3.88 ± 0.86	4.02 ± 0.84	0.14 ± 1.16	0.50	0.85	0.58

BMI, body mass index; TG, triglyceride; TC, total cholesterol; LDL-C, low density lipoprotein cholesterol; HDL-C, high density lipoprotein cholesterol; PYY, peptide YY; CCK, cholecystokinin; OXM, oxyntomodulin; ANGPTL4, angiopoietin-related 4, GPX, glutathione peroxidase. T-AOC: total antioxidant capacity. *p*_0_ indicates the differences before and after intervention within the group. *p*_1_ indicates the differences between groups after intervention. *p*_2_ indicates the differences between the changes. Individuals in the placebo (*n* = 40) and the synbiotic group (*n* = 40) were administered for BMI, body fat percentage, waist, hip, and soft lean mass. Individuals in the placebo (*n* = 35) and the synbiotic group (*n* = 32) were administered for serum lipids, hormone, and antioxidant indexes.

Table 3 shows the results of subgroup analysis, respectively, in obese individuals with abnormal LDL-C and those without dyslipidemia. In the obese individuals with abnormal LDL-C, significant reductions in body fat percentage (*p* = 0.02), hip circumference (*p* = 0.006), TG (*p* = 0.05) and LDL-C (*p* = 0.05) levels were observed in the synbiotic group while not in the placebo group. Moreover, the synbiotic group showed significantly lower post-intervention-LDL-C levels compared to the placebo group (*p* = 0.05), with a greater reduction in LDL-C (*p* = 0.04). However, in the obese individuals without dyslipidemia, except for reducing waist, the synbiotic intervention did not demonstrate any discernible superior effect compared to the placebo group, as evidenced by similar changes observed in various parameters following the intervention in both groups.

**Table 3:** Effect of synbiotics intervention on body composition of individuals of different subgroups.

	Placebo group		Synbiotic group			
	Change	*p*_0_	Change	*p*_0_	*p*_1_	*p*_2_
Subgroup1	(*n* = 9)		(*n* = 12)			
BMI (kg/m^2^)	−0.14 ± 0.63	0.34	−0.25 ± 0.82	0.29	0.72	0.48
Body fat percentage (%)	0.19 ± 2.14	0.81	−1.46 ± 1.86	0.02	0.87	0.08
Waist (cm)	0.33 ± 3.24	0.82	−2.69 ± 4.29	0.07	0.87	0.09
Hip (cm)	−1.33 ± 2.69	0.18	−3.69 ± 3.99	0.006	0.76	0.14
Soft lean mass (%)	0.87 ± 0.35	0.01	1.41 ± 1.74	0.01	0.99	0.56
TG (mmol/L)	0.22 ± 0.77	0.39	−0.17 ± 0.28	0.05	0.76	0.15
TC (mmol/L)	0.20 ± 0.60	0.31	−0.30 ± 0.64	0.12	0.16	0.07
LDL-C (mmol/L)	0.04 ± 0.44	0.81	−0.41 ± 0.48	0.01	0.05	0.04
HDL-C (mmol/L)	0.05 ± 0.11	0.24	−0.07 ± 0.14	0.09	0.49	0.09
Subgroup2	(*n* = 14)		(*n* = 16)			
BMI (kg/m^2^)	−0.09 ± 0.64	0.63	−0.30 ± 0.74	0.18	0.70	0.37
Body fat percentage (%)	−0.68 ± 1.23	0.02	−1.35 ± 1.81	0.004	0.98	0.19
Waist (cm)	−0.14 ± 5.03	0.92	−2.19 ± 3.02	0.01	0.21	0.08
Hip (cm)	−1.5 ± 3.36	0.05	−1.56 ± 1.67	0.002	0.81	0.95
Soft lean mass (%)	0.96 ± 1.34	0.03	1.26 ± 1.68	0.01	0.75	0.62
TG (mmol/L)	0.19 ± 0.37	0.03	0.01 ± 0.33	0.92	0.31	0.13
TC (mmol/L)	−0.03 ± 0.40	0.70	0.07 ± 0.36	0.47	0.27	0.43
LDL-C (mmol/L)	−0.05 ± 0.35	0.57	−0.02 ± 0.29	0.85	0.43	0.82
HDL-C (mmol/L)	−0.11 ± 0.14	0.001	0.002 ± 0.15	0.95	0.32	0.02

BMI, body mass index; TG, triglyceride; TC, total cholesterol; LDL-C, low density lipoprotein cholesterol; HDL-C, high density lipoprotein cholesterol; subgroup1, obese individuals with abnormal LDL-C; subgroup2, obese individuals without dyslipidemia. *p*_0_ indicates the differences before and after intervention within the group. *p*_1_ indicates the differences between groups after intervention. *p*_2_ indicates the differences between the changes.

### 3.3 Safety of synbiotics intervention on individuals with obesity

No adverse events were reported throughout the intervention. Following the intervention, there were no significant changes observed in liver (AST, ALT) and renal (UA, CR, UR) function indexes within both the placebo group and synbiotic group compared to baseline. Additionally, no obvious difference was found between the groups after the intervention, which indicates good safety of the synbiotics MN-Gup-GOS-XOS (Supplementary Table S1).

### 3.4 Dietary information of individuals with obesity

According to the dietary survey data, there were no significant differences in total energy, carbohydrate, fat, and protein intakes between the placebo and synbiotic group at baseline. After the intervention, dietary intakes did not change in both groups compared to baseline, and no significant difference was found between the two groups at post-intervention (Supplementary Table S2).

### 3.5 Effect of synbiotics intervention on gut microbiota of individuals with obesity

**Figure 1:**
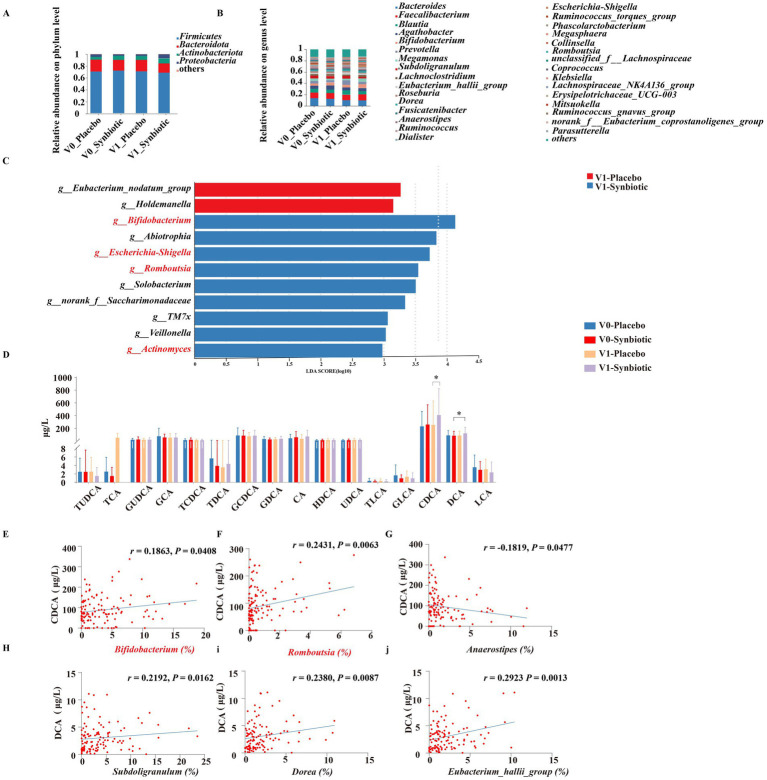
Effects of MN-Gup-GOS-XOS on gut microbiota and bile acids of individuals with obesity. V0: baseline, V1: post-intervention. **(A,B)** The average relative abundance of bacteria at the phylum and the genus level. **(C)** LEfSe analysis of gut microbiota at post-intervention. The length of the bar represents the log10 transformed LDA score. The threshold on the logarithmic LDA score for discriminative features was set to 2.0. **(D)** Bile acids in individuals at baseline and post-intervention. TUDCA, tauroursodeoxycholic acid; TCA, taurocholate acid; GUDCA, glycoursodeoxycholic acid; GCA, glycocholic acid; TCDCA, taurochenodeoxycholic acid; TDCA, taurodeoxycholic acid; GCDCA, glycochenodeoxycholic acid; GDCA, glycoursodeoxycholic acid; CA, cholic acid; HDCA, hyodeoxycholic acid; UDCA, ursodeoxycholic acid; TLCA, taurolithocholic acid; GLCA, glycolithocholic acid; CDCA, chenodeoxycholic acid; DCA, deoxycholic acid; LCA, lithocholic acid. **(E–J)** Pearson correlation heatmap of bile acids and gut microbiota.

As shown in Supplementary Figures S3A–D, 16S rDNA gene sequencing of feces in subjects were performed to explore the changes in gut microbiota. There was no significant difference in alpha diversity indicated by the Shannon, Simpson, Sobs and ACE index between the placebo and synbiotic groups at baseline and post-intervention. Beta diversity based on PCoA showed that the cluster of placebo group did not clearly separate from the cluster of the synbiotic group at baseline and post-intervention (Supplementary Figures S3E,F). At the level of phylum, the dominant microbes could be classified as four phyla, including *Firmicutes*, *Bacteroidetes*, *Actinobacteriota*, and *Proteobacteria* (Figure 1A). At the level of genus, the dominant bacterial including *Bacteroides*, *Faecalibacterium*, *Blautia*, *Agathobacter*, and so on (Figure 1B). LEfSe analysis revealed that *Eubacterium_nodatum_group* and *Holdemanella* were significantly enriched in the placebo group after intervention, while the synbiotic group enriched *Bifidobacterium*, *Abiotrophia*, *Escherichia-Shigella*, *Romboutsia*, *Solobacterium*, *TM7x*, *Veillonella*, and *Actinomyces* at the level of genus (Figure 1C). KEGG pathway analysis showed that the top three pathways of feces were carbohydrate metabolism, membrane transport, and amino acid metabolism (Supplementary Figure S3G).

### 3.6 Effect of synbiotics intervention on serum bile acids of individuals with obesity

The regulation of bile acid metabolism by MN-Gup-GOS-XOS is demonstrated in Figure 1D. Notably, the synbiotic group exhibited a significantly higher level of CDCA compared to the placebo group after intervention (*p* < 0.05). Additionally, a significant increase in DCA was observed in the synbiotic group compared to the baseline (*p* < 0.05). There were no significant changes or differences in other bile acid parameters within or between groups.

### 3.7 Correlation heatmap of bile acids and gut microbiota

The Person’s correlation analysis revealed a significant positive association between *Bifidobacterium* and *Romboutsia* with CDCA (Figures 1E,F), whereas *Anaerostipes* exhibited a significant negative correlation with CDCA (Figure 1G). Additionally, *Subdoligranulum*, *Dorea*, and *Eubacterium_hallii_group* demonstrated a significant positive correlation with DCA (Figures 1H–J).

### 3.8 Gut microbiota analysis in subgroups stratified by baseline serum lipid levels

**Figure 2:**
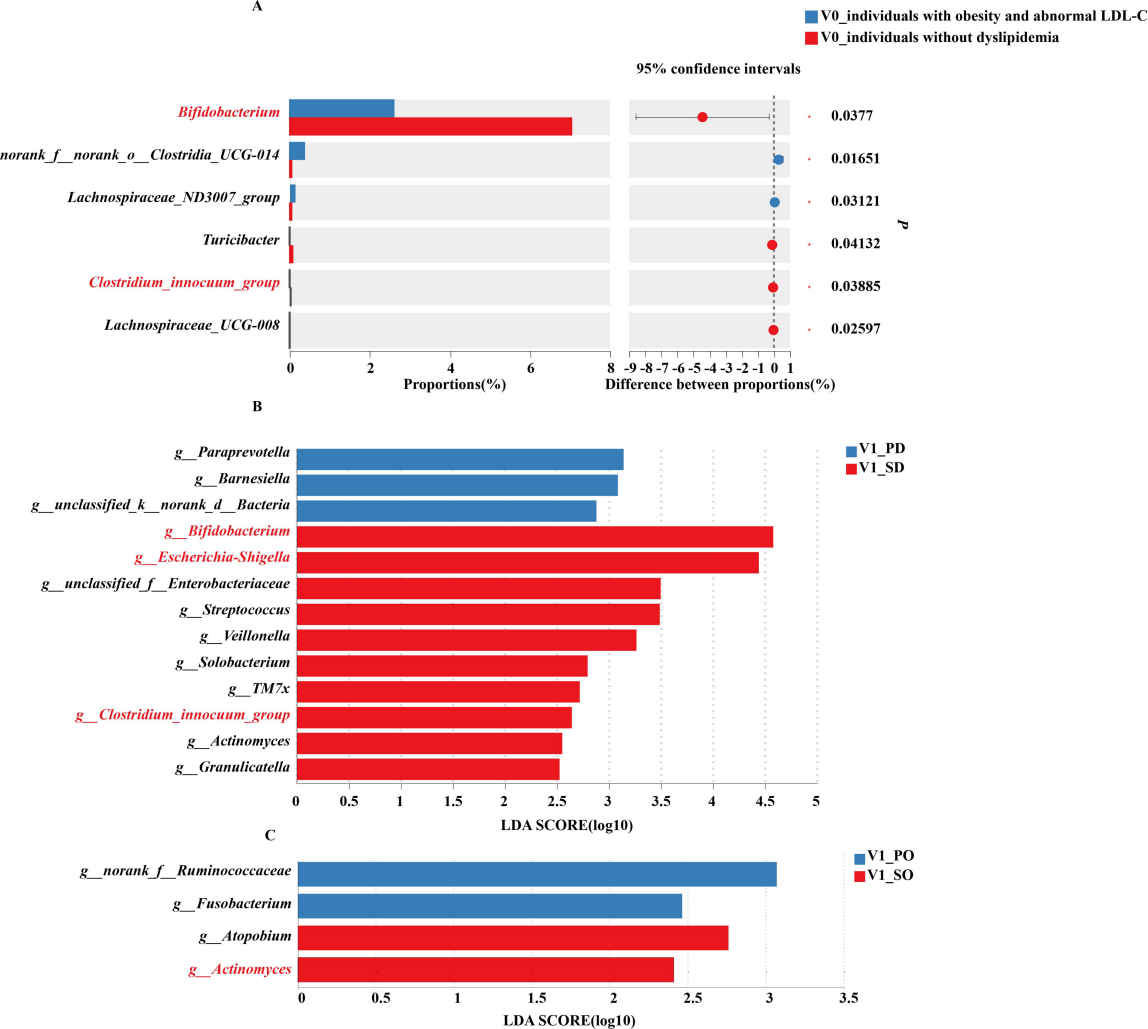
Gut microbiota of subgroup analysis. **(A)** Differences in gut microbiota (genus level) between obese individuals with abnormal LDL-C and obese individuals without dyslipidemia at baseline. **(B)** LEfSe analysis of gut microbiota in obese individuals with abnormal LDL-C at post-intervention. **(C)** LEfSe analysis of gut microbiota in obese individuals without dyslipidemia at post-intervention. PD, placebo group in obese individuals with abnormal LDL-C; SD, synbiotic group in obese individuals with abnormal LDL-C; PO, placebo group in obese individuals without dyslipidemia; SO, synbiotic group in obese individuals without dyslipidemia.

The results of gut microbiota analysis in subgroups stratified by baseline serum lipid levels are shown in the Figure 2.

At baseline, among the top 15% of genus abundance, significantly higher levels of *norank_f__norank_o__Clostridia_UCG-014* and *Lachnospiraceae_ND3007_group* were observed in obese individuals with abnormal LDL-C compared to those without dyslipidemia. Conversely, obese individuals without dyslipidemia showed a significant enrichment of *Bifidobacterium*, *Turicibacter*, *Clostridium_innocuum_group*, and *Lachnospiraceae_UCG-008* within their gut microbiota (Figure 2A).

After intervention, individuals with obesity and abnormal LDL-C exhibied significant enrichment of *Bifidobacterium*, *Escherichia-Shigella*, *Streptococcus*, *Veillonella*, *Solobacterium*, *TM7x*, *Clostridium_innocuum_group*, *Actinomyces*, and *Granulicatel* following synbiotics supplementation, while the placebo group enriched in *Paraprevotella* and *Barnesiella* (Figure 2B). In obese individuals without dyslipidemia, the placebo group exhibited a significant enrichment of *Fusobacterium*, whereas the synbiotic group demonstrated remarkable enrichment of *Atopobium* and *Actinomyces* (Figure 2C).

## 4 Discussion

This randomized, double-blind clinical trial showed that daily supplementation of the synbiotics MN-Gup-GOS-XOS for 12 weeks reduced body fat percentage, waist, serum LDL-C, and increased PYY, CCK, OXM, GSH in individuals with obesity. Additionally, the administration of MN-Gup-GOS-XOS resulted in an enhancement of certain gut microbiota and a significant increase in serum CDCA level in obese individuals. Correlation analysis revealed a significant association between certain gut microbiota and CDCA. Subgroup analysis demonstrated that MN-Gup-GOS-XOS had a more pronounced effect on obesity in individuals with abnormal LDL-C. Further analysis indicated that MN-Gup-GOS-XOS primarily improved the gut microbiota of obese individuals with abnormal LDL-C.

Improving obesity entails the crucial objective of reducing body fat and blood lipids. In this study, it was found that MN-Gup-GOS-XOS intervention could significantly reduce body fat percentage and waist, thereby indicating the potential of MN-Gup-GOS-XOS in enhancing body composition. Our study also demonstrated a significant reduction in serum LDL-C levels. Studies have shown that elevated serum LDL-C level is an important risk factor for cardiometabolic diseases in obese people (31). Therefore, it can be seen that MN-Gup-GOS-XOS in this study has the potential to reduce the risk of cardiometabolic disease in obese people. These results are consistent with the findings of other intervention studies, which demonstrated that synbiotic supplementation significantly reduced LDL-C levels in obese children or adults with overweight or obesity (32, 33). Besides, obesity usually leads to oxidative stress, and GSH helps to reduce the level of oxidative stress in the body (34). Our study indicates that after intervention with synbiotics, the serum GSH content of individuals significantly increased, suggesting that synbiotics can reduce oxidative stress levels in obese patients.

Studies have found that hormones play an important role in the regulation of lipid metabolism. For instance, PYY, CCK, OXM, and ANGPTL4 have been demonstrated to exert inhibitory effects on adipogenesis (15, 35–37). In this study, supplementation of MN-Gup-GOS-XOS increased the levels of PYY, CCK, and OXM in individuals with obesity, which provided a possible explanation for the mechanism of MN-Gup-GOS-XOS reducing LDL-C. Moreover, the metabolites such as bile acids related to the gut microbiota may exert a regulatory role on the aforementioned gastrointestinal hormones (38). Previous researches reported that DCA promotes PYY release in the colon and oral CDCA promoted the increase of OXM in human blood (11, 39). In this study, MN-Gup-GOS-XOS intervention increased the levels of CDCA, which may be the potential promoter of hormone release.

Furthermore, the efficacy of probiotics is inherently intertwined with the regulation of gut microbiota, which serves as the fundamental basis for metabolite production (40, 41). In this study, the relative abundance of *Bifidobacterium* in the synbiotic group was significantly higher compared to the placebo group at post-intervention, indicating that GOS and XOS facilitated the proliferation of *Bifidobacterium*. Furthermore, the synbiotic group exhibited enrichment in several bacteria such as *Escherichia-Shigella*, *Romboutsia*. *Solobacteriumm*, and *Actinomyces*, which have been proven to be beneficial for weight loss in previous animal and human studies (42–46). Additionally, Person’s correlation analysis revealed a significant positive association between *Bifidobacterium* and *Romboutsia* with CDCA, suggesting that MN-Gup-GOS-XOS may contribute to bile acid metabolism by promoting the proliferation of *Bifidobacterium* and *Romboutsia*.

Considering the observed beneficial impact of MN-Gup-GOS-XOS on LDL-C levels in individuals, we stratified the population into two subgroups: obese individuals with abnormal LDL-C and obese individuals without dyslipidemia. The intestine contains a large number of microorganisms that interact with the host (47). In this context, we observed that the MN-Gup-GOS-XOS supplement exerted a more favorable effect on serum LDL-C in obese individuals with abnormal LDL-C. In order to gain a more comprehensive understanding of this disparity in effects, we conducted an analysis of differential microbial communities. The relative abundances of *Bifidobacterium*, *Turicibacter*, *Clostridium_innocuum_group*, and *Lachnospiraceae_UCG-008* were significantly higher in obese individuals without dyslipidemia compared to those with abnormal LDL-C at baseline. The abundance of *Bifidobacterium* has been found to have a negative correlation with LDL-C (48). Furthermore, *Turicibacter* was found to increase with weight loss in overweight men (49). Additionally, *Clostridium_innocuum_group* has been identified as a beneficial bacterium associated with lipid metabolism (50). However, gut microbiota of obese individuals with abnormal LDL-C exhibited an enrichment of *Lachnospiraceae_ND3007_group*, which has been previously identified as a biomarker for various diseases in scientific literatures (51, 52). These findings implied, unsurprisingly, that dyslipidemia-free obese individuals harbored a gut microbiota profile indicative of relatively favorable health status. After 12 weeks of synbiotic consumption, the abundance of *Bifidobacterium* and *Clostridium_innocuum_group* in obese individuals with abnormal LDL-C levels exhibited a significantly higher increase compared to the placebo group. These findings suggest that MN-Gup-GOS-XOS can significantly promote the proliferation of beneficial bacteria.

The strengths of this study lie in the inclusion of a novel probiotic strain, MN-Gup, and its synbiotics. Additionally, our findings demonstrate that synbiotics exhibit superior efficacy in ameliorating obesity and lipid metabolism abnormalities among individuals with elevated LDL-C levels. The study also presents certain limitations. Firstly, the direct correlation between gut microbiota and bile acids remains unclarified, while the precise mechanism underlying the impact of MN-Gup-GOS-XOS has not been fully elucidated. Secondly, the examination of beneficial bacteria promotion on CDCA was not conducted, leaving room for future verification through microbiota transplantation. Thirdly, this study only detected bile acids in serum, and future research can further explore the relationship between bile acids and obesity by detecting bile acids in feces. Fourthly, it is important to note that the findings of this study are limited in their applicability to individuals with obesity. Therefore, further recruitment of subjects with different types of obesity is recommended to explore the effects of synbiotics.

In conclusion, a 12-week supplementation of MN-Gup-GOS-XOS demonstrated significant reduction in body fat percentage, waist, and serum LDL-C among obese individuals, accompanied by alterations in gut microbiota (*Bifidobacterium* and *Romboutsia*), bile acid (CDCA), hormones (PYY, CCK, and OXM), and GSH. Furthermore, MN-Gup-GOS-XOS seemed to have a more favorable impact on individuals with abnormal LDL-C levels, which was further supported by the modifications seen in gut microbiota. However, the exact mechanism requires further validation through subsequent studies.

## Data Availability

The datasets presented in this study can be found in online repositories. The names of the repository/repositories and accession number(s) can be found in the article/Supplementary material.
