# Supplementary material for: Effect of synbiotic supplementation on obesity and gut microbiota in obese adults: a double-blind randomized controlled trial
Source: Front Nutr. 2024 Nov 27;11:1510318. doi: 10.3389/fnut.2024.1510318 (PMC11633458; doi:10.3389/fnut.2024.1510318)
Supplement: Supplementary file 1 [file Data_Sheet_1.docx]

Supplementary Material

**Table S1** Biochemical markers of individuals with obesity

|  | Placebo (n = 40) | |  | Synbiotic (n = 40) | |  |  |
| --- | --- | --- | --- | --- | --- | --- | --- |
|  | Baseline | Post-intervention | *P_0_* | Baseline | Post-intervention | *P_0_* | *P_1_* |
| AST (mmol/L) | 21.35 (18.00, 24.50) | 22.50 (16.90, 33.50) | 0.52 | 19.85 (17.30, 23.35) | 20.10 (16.75, 23.15) | 0.73 | 0.20 |
| ALT (mmol/L) | 19.00 (13.80, 31.10) | 16.90 (11.60, 42.00) | 0.92 | 20.70 (13.25, 30.30) | 18.05 (11.65, 33.60) | 0.16 | 0.67 |
| UA (μmol/L) | 334.55 ± 77.02 | 337.92 ± 84.22 | 0.88 | 352.97 ± 77.84 | 361.03 ± 77.59 | 0.33 | 0.23 |
| CR (μmol/L) | 72.83 ± 11.02 | 73.21 ± 11.55 | 0.89 | 72.07 ± 12.65 | 71.69 ± 13.86 | 0.72 | 0.60 |
| UR (mmol/L) | 4.64 ± 0.78 | 4.85 ± 0.81 | 0.16 | 4.73 ± 1.15 | 4.75 ± 0.90 | 0.79 | 0.62 |

AST: aspartate aminotransferase, ALT: alanine aminotransferase, UA: uric acid, CR: creatinine, UR: urea. *P_0_* indicates the differences before and after intervention within the group. *P_1_* indicates the differences between groups after intervention.

**Table S2** Dietary information at baseline and post-intervention

|  | Placebo (n = 40) | |  | Synbiotic (n = 40) | |  |  |
| --- | --- | --- | --- | --- | --- | --- | --- |
|  | Baseline | Post-intervention | *P_0_* | Baseline | Post-intervention | *P_0_* | *P_1_* |
| Energy (kcal) | 1834.79 ± 295.16 | 1844.36 ± 166.04 | 0.86 | 1863.25 ± 179.56 | 1888.36 ± 374.85 | 0.71 | 0.50 |
| Carbohydrate (g) | 310.36 ± 79.43 | 317.60 ± 49.80 | 0.56 | 306.23 ± 62.67 | 332.33 ± 79.48 | 0.11 | 0.35 |
| Protein (g) | 76.34 ± 31.92 | 74.72 ± 22.42 | 0.77 | 78.89 ± 23.92 | 73.89 ± 22.81 | 0.32 | 0.77 |
| Fat (g) | 28.14 ± 23.12 | 26.81 ± 14.52 | 0.72 | 32.50 ± 19.40 | 25.63 ± 14.64 | 0.10 | 0.72 |

*P_0_* indicates the differences before and after intervention within the group. *P_1_* indicates the differences between groups after intervention.


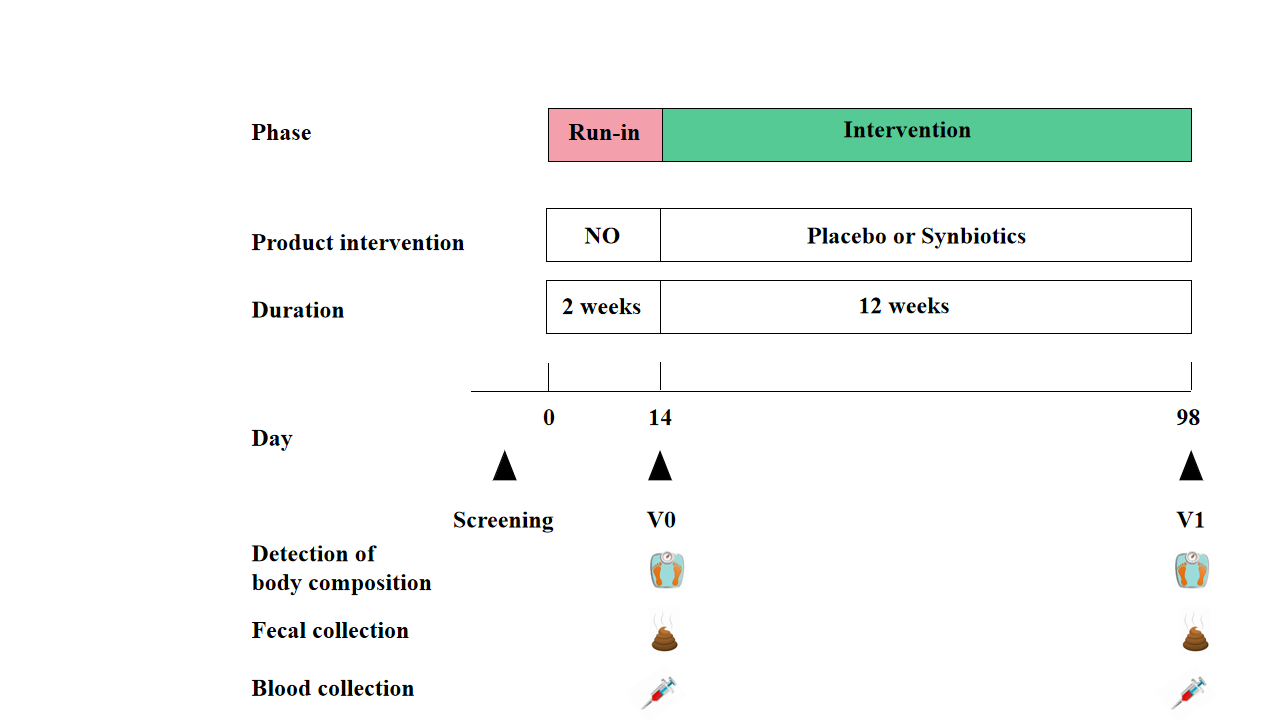


**Figure S1** Study design. Synbiotics: lyophilized powder containing Bifidobacterium animalis subsp. lactis MN-Gup galacto-oligosaccharides, and xylo-oligosaccharides. Placebo: maltodextrin. V0: Baseline, V1: Post-intervention


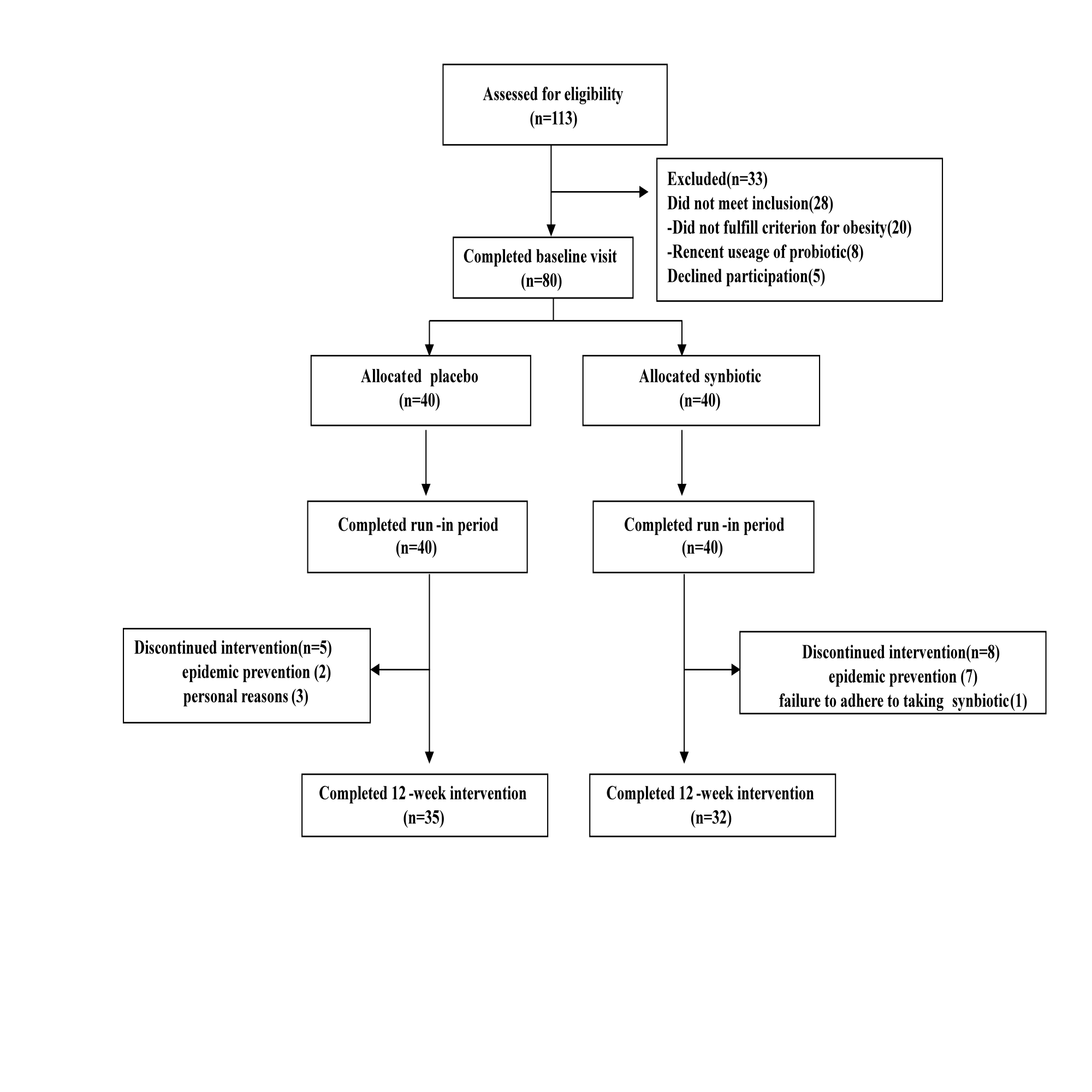


**Figure S2** Trial profile


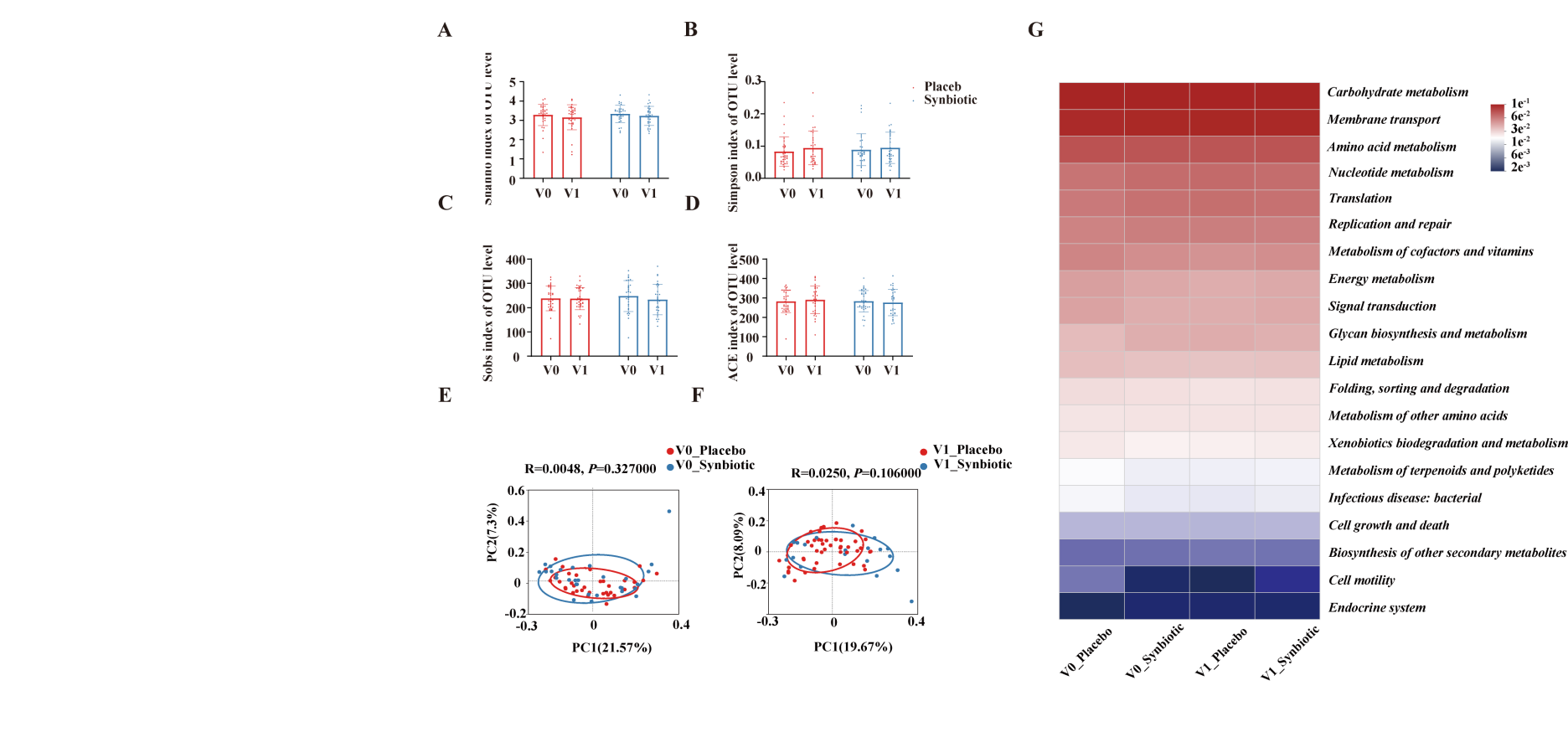


**Figure S3** Gut microbiota of individuals with obesity. (a)- (d) α diversity. (e)-(f) β diversity. PCoA based on bacterial operational taxonomic units (OTUs) using unweighted-unifrac calculation. ANOSIM was used to analyze the similarity analysis, and R value > 0 indicates that the difference between groups is greater than the difference in groups, and P < 0.05 indicates significant differences. (g) LEfSe analysis of Placebo group. ACE: Abundance-based coverage estimators. V0: Baseline, V1: Post-intervention.
